# Localization of the Swainsonine-Producing Chaetothyriales Symbiont in the Seed and Shoot Apical Meristem in Its Host *Ipomoea carnea*

**DOI:** 10.3390/microorganisms10030545

**Published:** 2022-03-02

**Authors:** Marwa Neyaz, Dale R. Gardner, Rebecca Creamer, Daniel Cook

**Affiliations:** 1 Plant and Environmental Sciences, New Mexico State University, Las Cruces, NM 88003, USA; marwane@nmsu.edu; 2 Poisonous Plant Research Laboratory, US Department of Agriculture, Logan, UT 84321, USA; dale.gardner@usda.gov; 3 Department of Entomology, Plant Pathology, and Weed Science, New Mexico State University, Las Cruces, NM 88003, USA; creamer@nmsu.edu

**Keywords:** swainsonine, Chaetothyriales, *Ipomoea*, symbiont, microscopy, shoot apical meristem

## Abstract

Several species of fungi from the orders Chaetothyriales and Pleosporales have been reported to produce swainsonine and be associated as symbionts with plants of the Convolvulaceae and Fabaceae, respectively. An endosymbiont belonging to the Chaetothyriales produces swainsonine and grows as an epibiont on the adaxial leaf surfaces of *Ipomoea carnea*, but how the symbiont passes through plant growth and development is unknown. Herein, different types of microscopy were used to localize the symbiont in seeds and in cross sections of plant parts. The symbiont was found in several tissues including the hilum, the sclereids, and the hypocotyl of seeds. In five-day old seedlings and mature plants, the symbiont was found in the shoot apical meristem (SAM) and the adaxial surface of immature folded leaves. The mycelia generally formed a close association with peltate glandular trichomes. This report provides further data explaining the relationship between the seed transmitted Chaetothyriales symbiont and *Ipomoea carnea*. These results provide a possible explanation for how this symbiont, and others like *Periglandula* may persist and are transmitted over time.

## 1. Introduction

Plants are exposed to variety of microorganisms during their life cycle [1]. These interactions can be classified into three categories: parasitic, commensalistic, and mutualistic. Vertically or seed transmitted microorganisms may play critical roles in life cycles of many organisms. Generally, vertically transmitted symbionts are thought to improve the fitness of their host and subsequently benefit themselves as they are often completely dependent upon the host for transmission [2,3]. Symbionts that are vertically transmitted are associated with a few select plant families including the Poaceae, Fabaceae, and Convolvulaceae [4]. A notable characteristic of many plant–symbiont fungal associations, including vertical transmission and a narrow host range with specific plant families, is the production of bioactive secondary metabolites. Understanding the association of these heritable fungal symbionts may provide insights into the evolution and ecology of the host and symbiont.

Swainsonine, an indolizidine alkaloid, is the toxic principle in several plant species worldwide and causes severe toxicosis in livestock grazing these plants [5,6,7,8,9]. Swainsonine occurs sporadically in three diverse plant families: the Convolvulaceae, Fabaceae, and Malvaceae [10]. Swainsonine, an alpha-mannosidase and mannosidase II inhibitor, alters glycoprotein processing and causes lysosomal storage disease [5,11,12]. Consumption of swainsonine-containing plants by grazing animals leads to a chronic disease characterized by weight loss, depression, altered behavior, decreased libido, infertility, and death, which are estimated to cause tens of millions of dollars in livestock losses annually [13].

All swainsonine-containing plant taxa investigated to date are associated with seed-transmitted fungal symbionts that produce swainsonine. Legumes containing swainsonine are associated with a fungal symbiont belonging to *Alternaria* section *Undifilum* species (Pleosporales) [14,15,16,17], while *Ipomoea carnea*, a member of the Convolvulaceae, is associated with a symbiont belonging to the order Chaetothyriales [18]. In summary, the two disjunct plant groups that contain swainsonine, legumes and morning glories, are associated with two disjunct groups of swainsonine-producing seed transmitted fungal symbionts, the Pleosporales and Chaetothyriales.

The swainsonine-producing seed-transmitted symbionts associated with the legumes and morning glories can be cultured from leaves and seeds. These same taxa that lack the respective corresponding symbionts also lack swainsonine [17,18,19]. The seed-transmitted symbionts associated with legumes grow between cells and are associated with the seed coat [19,20,21]. In contrast, the seed-transmitted symbiont in *Ipomoea carnea* grows as an epibiont on the adaxial (upper) leaf surfaces but not on the abaxial surface. Fungal mycelia were not observed on any other plant surfaces, or cross sections of petioles and stems of symbiont-containing plants [22]. Due to the restricted tissues where this symbiont is present, it is not known how the symbiont colonizes *Ipomoea carnea* plants to infect new growth and future seeds.

The objective of the present study was to determine how the symbiont is passed through plant growth and development. Cook et al. [18] demonstrated that the Chaetothyriales symbiont can be cultured from seeds that have been surface sterilized and that the symbiont is able to recolonize newly developing leaf surfaces from plants clipped back to woody stems, suggesting that the symbiont is found within plant tissues. Here, we investigate seeds and vegetative tissues, including meristematic regions that contain swainsonine and do not contain swainsonine, for the symbiont to further understand the host–symbiont association. The study of plant–fungal interactions is key to understanding the evolutionary association among hosts and symbionts.

## 2. Materials and Method

### 2.1. Seeds and Plants

All seeds and/or plants were defined as containing the symbiont (positive) or lacking the symbiont (negative) based upon the presence or absence of swainsonine. Swainsonine was detected using methods previously described [23,24].

*Ipomoea carnea* (Jacq.) subsp. *fistulosa* (Mart. ex Choisy) seeds (positive) were collected in April 2011 near the veterinary hospital of the University of Campina Grande, Campus of Patos in the city of Patos, Paraiba, Brasil (7°04′02″ S 37°16′53″ W) (UTC 00260470). *Ipomoea carnea* seeds lacking the symbiont (negative) were purchased from Onalee’s seeds (https://www.onalee.com/ (accessed on 25 February 2022). *Ipomoea riedelli* seeds (positive) were collected in the municipality of Zabelé, state of Paraíba, Brazil [25].

To eliminate the seed-associated fungal symbiont, *Ipomoea carnea* seeds (*n* = 15) were scarified and imbibed overnight in a 0.9% pyraclostrobin (BASF, Research Triangle Park, NC, USA) solution. Pyraclostrobin, a strobilurin class fungicide, has a mode of action shown to be effective against a broad range of fungal species including many Ascomycetes. Non-treated (positive) and treated seeds (negative) were potted, and the resulting plants were grown in the greenhouse. Mature plants (>5 years old) derived from the above-mentioned seeds were grown in a greenhouse with day/night temperatures of 25 °C/20 °C and a 16 h photoperiod, fertilized monthly during the growing season with all-purpose plant food (Peters professional, Summerville, SC, USA) 1.3 mL per L of water, and pruned back by clipping the tall shoots and branches. Five-day-old seedlings were generated from positive and negative seeds, respectively. Seeds were cleaned from seed hair, surface sterilized, scarified with medium grit sandpaper, and soaked in water for 24 h prior to planting in 6 × 6 cm pots. Seeds were potted and incubated in a growth chamber under incandescent light at 25 °C. At day five, plants were extracted out of the soil, rinsed with water, and processed for additional studies.

Leaves from positive plants were excised from the plant. Using a dissecting microscope, tissues from the margin leaf not colonized by the symbiont were excised with sterilized razor blades, and the corresponding tissues containing the symbiont were air dried, and analyzed for swainsonine.

### 2.2. Stereofluorescence Microscopy and Laser Scanning Confocal Microscopy

Whole seeds (positive and negative), specifically the hilum and longitudinal sections, were examined with a model M165FC stereofluorescence microscope SFM and a model TCS SP5 II laser scanning confocal microscope LSCM (Leica Microsystems, Buffalo Grove, IL and Exton, PA, USA). Embryos were imaged by reflection using a model LED 5000 RL white light and epifluorescence from a metal halide lamp with filter sets for violet, red, blue, and green excitation to visualize superficial structural features. All observations in this study were made for at least 20 seeds and plant parts. Enhancement of brightness and/or contrast were applied to some of the images mainly to show fungal structure when needed, and no background or spot editing was applied.

The hilum was examined in intact whole seeds (positive and negative) by confocal microscopy as follows: seed hair was removed from seeds with a wet paper towel, fixed in 2.5% glutaraldehyde for at least 5 days prior to microscopy, and rinsed with 1× phosphate buffered saline (PBS) prior examination. The seeds were mounted in modified plastic petri dishes with glass bottom cover glasses (MatTek Corp., Ashland, MA, USA) in a universal holding frame of an inverted microscope base. Seeds were propped up using 2% agarose blocks on each side of the seed, which acted as stoppers as seeds did not lay level on the dish due to their shape. This allowed clear imaging of the hilum when using the confocal microscope.

The seed coat, the sclereid tissue, and the hypocotyl from seeds (positive and negative) were examined by confocal microscopy as follows: positive and negative seeds were cleaned from hairs, surface sterilized by soaking in 70% ethanol for 3 min and 10% bleach for 2 min, rinsed with distilled water (DIH_2_O) for 1 min, scarified with medium grit, and soaked in DIH_2_O for 24 h. Samples were then fixed in 2.5% glutaraldehyde for 5–10 days and subsequently rinsed in 1× PBS prior to examination. Razor blades were sterilized using 95% ethanol to perform longitudinal sectioning of the respective tissues.

Samples of the shoot apical meristem (SAM), petiole, and dormant buds were prepared from 5-day-old seedlings, while samples of the ovules and anthers were obtained from mature plants. All samples were fixed in 2.5% glutaraldehyde for 5–10 days prior to preparing cross sections and subsequent microscopic examination.

All samples were examined using a water-immersion 25× (n.a. 0.9) long working distance objective lens. Fluorescence was excited with a 405 nm diode laser. To capture plant and fungal autofluorescence, emission was collected in three image channels, blue (410–490 nm), green (500–580 nm), and red (600–720 nm). Stacks of optical sections (~20–40 μm deep) were collected and displayed as overlays of the three image channels as maximum projection images.

### 2.3. Scanning Electron Microscopy

The hilum was examined from seeds (positive and negative) that were not scarified or soaked in water, only de-haired and fixed in 2.5% glutaraldehyde buffered with 0.1 M imidazole-HCl at pH 7.2 for 3 days at room temperature. The embryo, including the sclereids and the hypocotyl from seeds (positive and negative) as well as SAM tissues, was prepared as follows: seeds were scarified with medium grit and soaked in water for 24 h, and SAM tissues were fixed in 2.5% glutaraldehyde for 3 days at room temperature. Following fixation, samples were rinsed with 1× PBS buffer for 10 min and subjected to a series of ethanol dehydration: 50%, 70%, 80%, and 100% ethanol, a 1:1 ethanol and hexamethyldisilazane (HMDS) solution, and HMDS absolute for 15–20 min for each step at room temperature. Samples then were laid on filter paper in mini glass petri dish with absolute HDMS overnight [26,27]. Samples were mounted on aluminum stubs with carbon adhesive tape and colloidal silver paint (Electron Microscopy Sciences, Hatboro, PA, USA) followed by sputter coating with gold-palladium using a Desk IV thin film coating system (Denton Vacuum, Moorestown, NJ, USA). Topographical digital images were collected using a model S-3400N II scanning electron microscope (Hitachi High-Technologies, Dallas, TX, USA) operated in the high vacuum, secondary electron imaging mode.

### 2.4. Fluorescent In Situ Hybridization (FISH)

The hilum from positive and negative seeds and SAM from 5-day-old positive and negative seedlings was examined by fluorescent in situ hybridization (FISH). Seeds were scarified with medium grit, and soaked in water for 12 h, and cross-sectioned, and the hilum was separated from the seed to minimize sample size. The general steps of FISH were performed as described in [28]. Hilum and SAM tissues were soaked separately in 4% paraformaldehyde in PBS (4.75% DI H_2_O, 2% paraformaldehyde, and 0.75% PBS) and gently mixed for 2 h, then incubated at 5 °C overnight or until use. Samples were washed in 1× PBS buffer for 10 min, permeabilized by incubating in 0.1% Triton X-100 in PBS buffer for 10 min, and washed twice with 1× PBS for 10 min. The hybridization mixture was added (10 µL of 20% dextran sulfate, 10 µL of 1.25× concentrate hybridization buffer ENZO Life Sciences Inc., Farmingdale, NY, and 10 µL of *swnK* forward probe [29] ATTO590 Integrated DAN Technologies, Coralville, IA, USA) to the samples and incubated in a water bath at 100 °C for 5 min, then incubated on ice for 10 min. The hybridization mixture was then removed from the tubes, and samples were rinsed twice with 2× saline-sodium citrate (SSC) buffer for 20 min each, followed by a third wash with 1× SSC for 10 min. Wash buffer was removed, and samples were rinsed with 1× PBS for 2 min, followed by water rinse for 2 min. Samples were examined using the confocal microscope, and emission was collected in two image channels: cyan (blue and green) to capture the autofluorescence of plant cells and red (650–720 nm) to capture the fungal mycelia hybridized with the *swnK* probe (ATTO590 Integrated DAN Technologies, Coralville, IA, USA), and the probe was excited at 594 nm and emitted at 660 nm in red.

## 3. Results

### 3.1. Seeds

**Figure 1:**
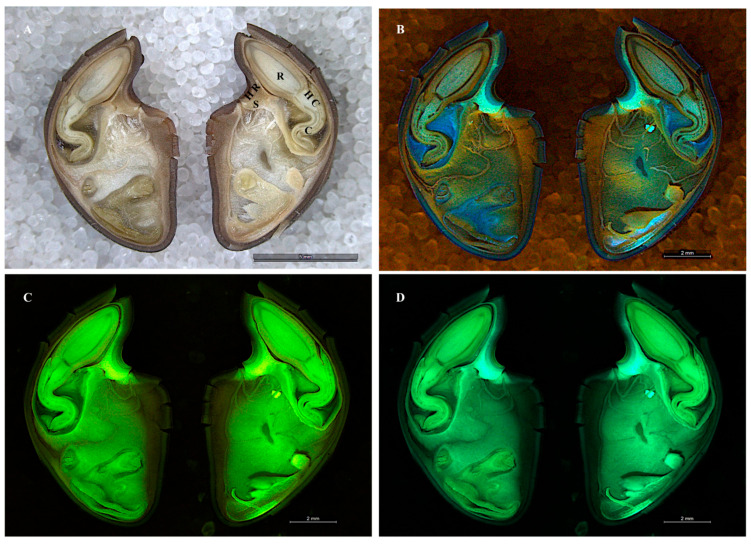
Stereofluorescence microscopy images of a longitudinal section of a hydrated positive *Ipomoea carnea* seed shown as a point of reference with the following tissues labeled: hilum and the hilum ridge labeled (HR), the sclereids (S), the hypocotyl (HC), the radicle (R), and the cotyledons (C). Images captured by the reflection of white light (**A**) scale bar—5 mm, violet light (**B**) scale bar—2 mm, red light (**C**) scale bar—2 mm, and GFP1 light (**D**) scale bar—2 mm.

**Figure 2:**
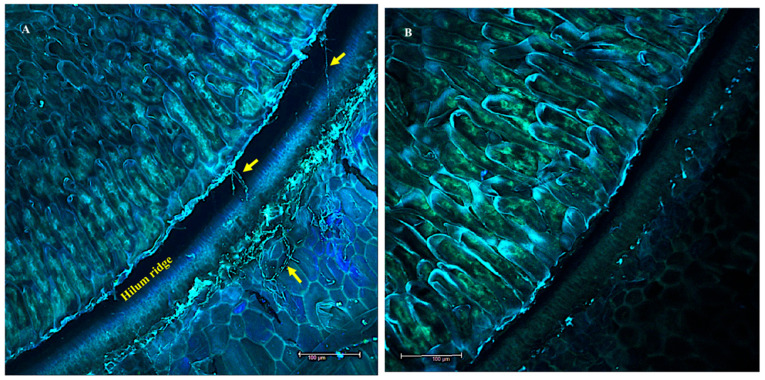
Fluorescent laser scanning confocal microscope images of the hilum from *Ipomoea carnea* seeds; positive (**A**) and negative (**B**). Cyan (blue + green) autofluorescence of loose network of mycelia around the hilum and in the hilum crevice indicated by the arrows. No mycelia were observed in the hilum of negative seeds (**B**). Scale bar—100 μm.

**Figure 3:**
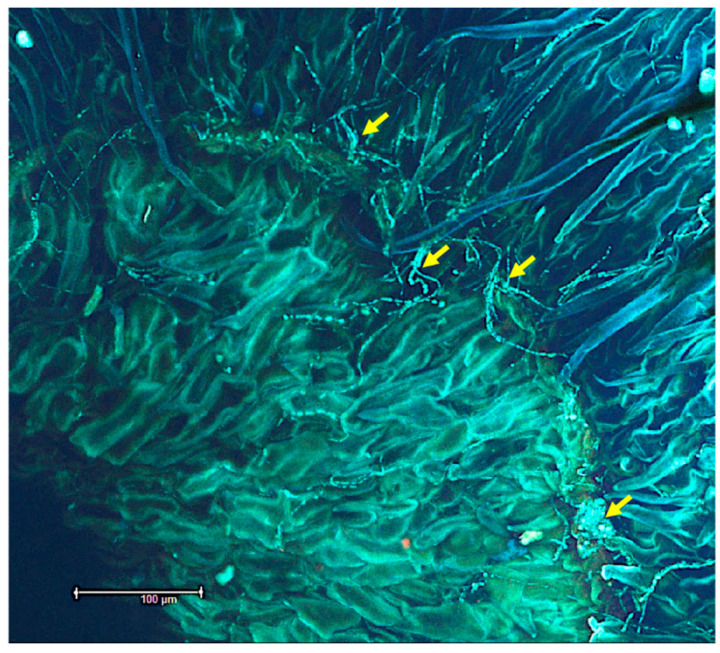
Fluorescent laser scanning confocal image shows fine threads of mycelia of the Chaetothyriales symbiont around the closed seed hilum of *Ipomoea riedelii* indicated by the arrows. Scale bar— 100 μm.

**Figure 4:**
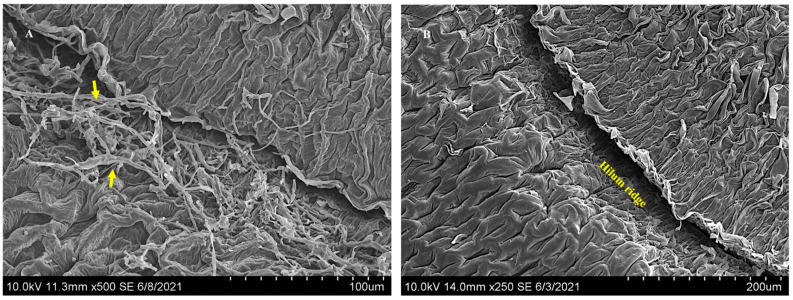
Scanning electron microscope images illustrating a topographical view of the hilum from *Ipomoea carnea* seeds; positive (**A**) and negative (**B**). Extensive network of mycelia observed on the positive hilum ridge indicated with arrows. No mycelia were observed in the hilum of negative seeds.

Longitudinal sections of swainsonine positive *Ipomoea carnea* seeds were examined by stereofluorescence microscopy; images are shown as a point of reference with the hilum and the hilum ridge, the sclereids, the hypocotyl, the radicle, and the cotyledons labeled (Figure 1). The Chaetothyriales symbiont was located in *I. carnea* positive seeds at the hilum ridge and in the open crevice of the hilum, forming a mycelial network that fluoresced in cyan (blue-green) (Figure 2A). No mycelia were detected in *I. carnea* negative seeds at the hilum ridge and in the open crevice of the hilum (Figure 2B). Similar to *I. carnea*, fungal mycelia were detected in the seeds of *I. riedelii*, another swainsonine-containing species, by confocal microscopy at the hilum ridge and in the open crevice of the hilum (Figure 3). SEM micrographs showed a similar location of the Chaetothyriales symbiont in *I. carnea* positive seeds, where it formed an extensive network at the bottom half of the hilum lip (Figure 4A). No mycelia were detected in negative seeds near or around the hilum (Figure 4B).

**Figure 5:**
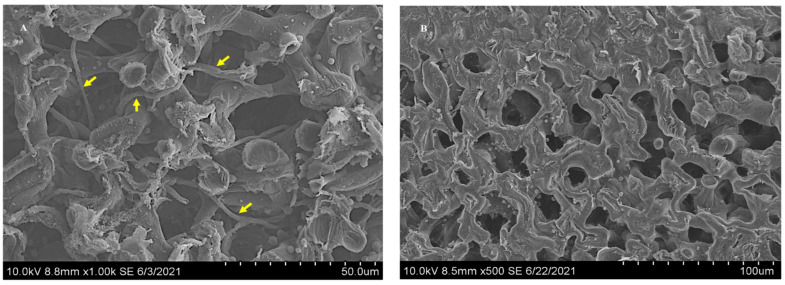
Scanning electron microscope images of fungal mycelia tangled around the sclereids tissue of a positive *Ipomoea carnea* seed indicated by the arrows (**A**), and absence of mycelia in *I. carnea* negative seed sclereids (**B**).

**Figure 6:**
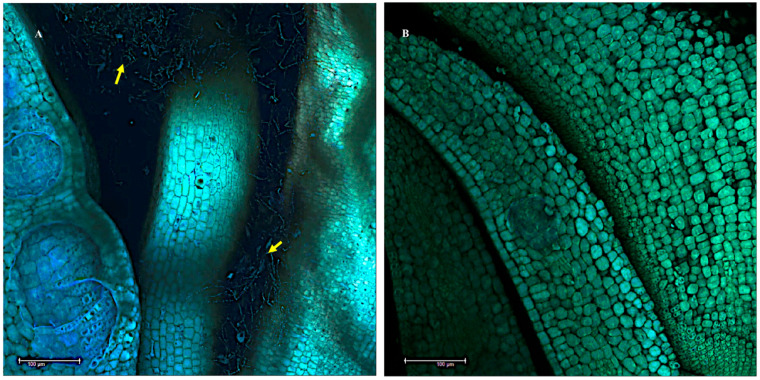
Fluorescent laser scanning confocal microscope images showing massive network of mycelia colonizing the intercellular spaces in the hypocotyl region of positive *Ipomoea carnea* seeds indicated by the arrows (**A**). No colonization of mycelia was observed in the hypocotyl region from a negative seed (**B**). Scale bar—100 μm.

**Figure 7:**
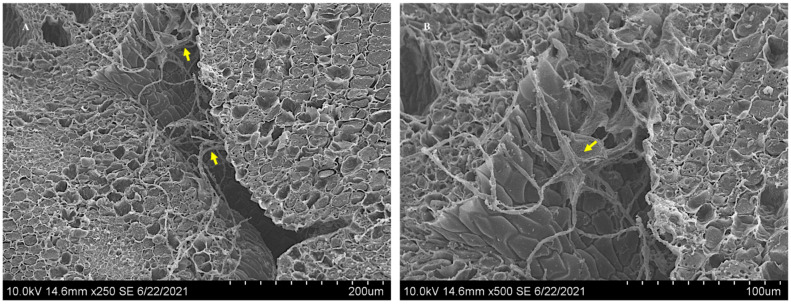
Scanning electron microscope images illustrating fungal growth between the gaps of the hypocotyl and the cotyledons of positive *Ipomoea carnea* seed indicated by the arrows (**A**). The mycelia that appears on the top surface is an artifact of sectioning. A magnified image ×500 of the hypocotyl region shows that mycelia are restricted to the gaps and do not penetrate the plant cells (**B**).

Mycelia were also found to be tangled around the sclereids of hydrated *I. carnea* positive seeds by SEM (Figure 5A), while Figure 5B illustrates the complete absence of symbiont mycelia in the sclereids of hydrated negative seeds. Fungal mycelia were detected by confocal microscopy colonizing the intercellular spaces of the hypocotyl region between plant tissues in hydrated positive seeds (Figure 6A); in contrast, no mycelia were detected near the hypocotyl of hydrated negative seeds (Figure 6B). Investigation of mycelial growth in the hypocotyl using SEM showed the mycelia restricted to the gaps between the radicle and the cotyledons in hydrated positive seeds; notably, no penetration of mycelia into the plant cells was observed (Figure 7). In the hydrated seeds, mycelia were only observed in the hypocotyl, immediately above the radicle, and under the cotyledons (Supplemental Figure S1A). No mycelia were detected in other regions of the cotyledons (Supplemental Figure S1B–D). Lastly, the mycelia did not appear to invade any section of the radicle germinated from positive seeds (Supplemental Figure S2).

### 3.2. The Shoot Apical Meristem SAM

**Figure 8:**
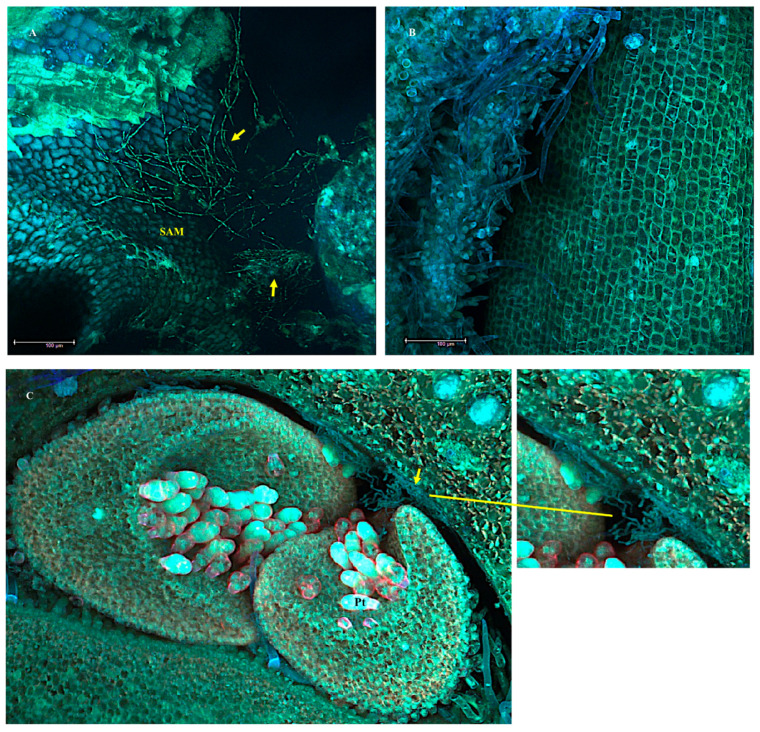
Fluorescent laser scanning confocal microscope images showing network of mycelia in the shoot apical meristem (SAM) of a 5-day-old *I. carnea* positive plant colonizing intercellular spaces indicated by arrows (**A**), and no evidence of mycelia in the SAM of a 5-day-old negative plant (**B**). Mycelia in the SAM of 5+ year-old positive plant indicated by arrows (**C**). Peltate glandular trichome (Pt). Scale bar—100 μm.

**Figure 9:**
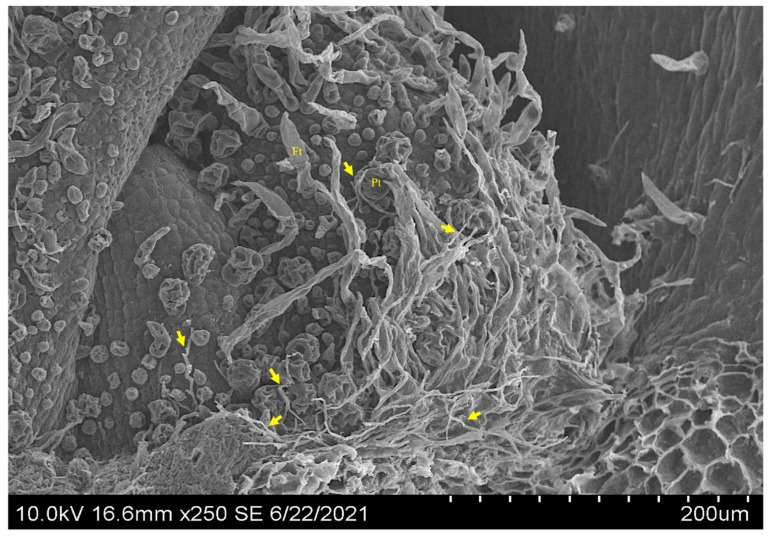
Scanning electron microscope images showing growth of the mycelia in the longitudinal section of the shoot apical meristem (SAM) of a 5-day-old *I. carnea* positive plant. Arrows point at mycelia in the SAM forming close association with peltate glandular trichomes (Pt) and filiform trichomes (Ft).

Using confocal microscopy, symbiont mycelia were localized abundantly near the SAM of 5-day-old *I. carnea* seedlings colonizing intercellular spaces (Figure 8A) and were not observed near the SAM of 5-day-old negative plants (Figure 8B). Similarly, mycelia were localized near the SAM of mature positive plants and formed a close association with peltate glandular trichomes (Figure 8C). SEM images confirmed the presence of extensive colonization of the symbiont near the SAM of 5-day-old plants (Figure 9) that had been shown by confocal microscopy. Figure 9 illustrates detailed colonization of the symbiont near the SAM and the close interaction with peltate trichomes indicated by red arrows.

### 3.3. Leaves and Other Plant Parts

**Figure 10:**
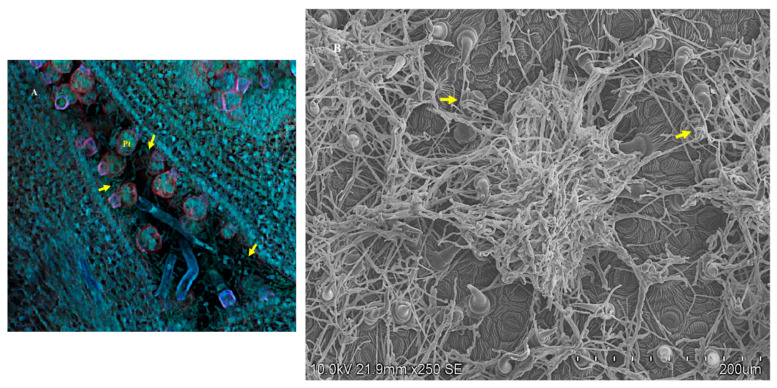
Fluorescent laser scanning confocal microscope of a longitudinal section of the adaxial surface of an *I. carnea* positive closed leaf from a mature plant (**A**) forming close contact with the peltate glandular trichomes (Pt) (scale bar—100 μm). Scanning electron microscope showing topographical view of the mycelia on expanded leaf anchored to filiform trichomes and on top peltate gradual trichomes indicated by the arrows (**B**).

Confocal images of the adaxial surface of an immature closed leaf showed the symbiont colonizing the *I. carnea* leaf surface with a close association with the peltate and filiform trichomes (Figure 10A). In expanded leaves, fungal mycelia were found on the adaxial leaf surface, apparently anchored to filiform trichomes and peltate trichomes (Figure 10B). No mycelia were detected on these surfaces from leaves that did not contain swainsonine (data not shown).

Mycelia were also identified on surfaces of the dormant bud sheath wrapped around peltate trichomes (Supplemental Figure S3A). Mycelia were not detected in the cross sections of the seed coat (Supplemental Figure S3B), the anther (Supplemental Figure S3C), or the vascular bundle (Supplemental Figure S3D).

### 3.4. Fluorescent In Situ Hybridization (FISH)

**Figure 11:**
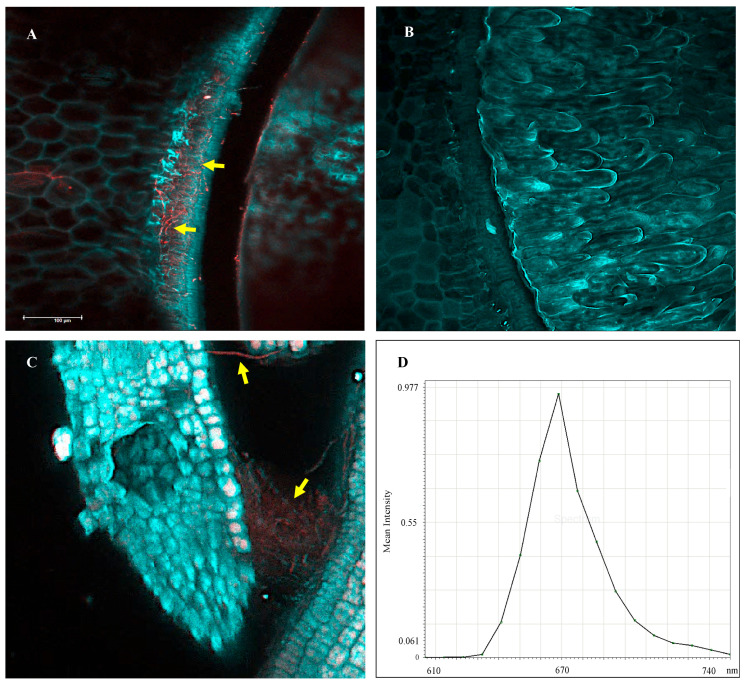
Fluorescent in situ hybridization (FISH) applied on an *Ipomoea carnea* positive seed hilum (**A**), negative seed hilum (**B**), and positive hypocotyl (**C**). Probe attachment to fungal mycelia emitted in red and indicated by arrows in (**A**,**C**). Scale bar—100 μm. Samples were examined using the confocal microscope, and emission was collected in two image channels: cyan (blue and green) to capture autofluorescence of plant cells and red (650–720 nm) to capture the fungal mycelia hybridized with *swnK* probe. Probe was excited at 594 nm, and maximum emission was at ~670 nm in red (**D**).

FISH was performed on the hilum of a *I. carnea* positive seed as an additional method to confirm symbiont identity (Figure 11A), while negative seeds were used as control (Figure 11B). Red fluorescence was observed around the hilum and in the crevice on the hilum of positive seeds. In the embryo, red fluorescence was observed in the gap between the hypocotyl and the cotyledon (Figure 11C). Maximum emission of the *swnK* probe (ATTO690) was observed at wavelength ~670 nm (Figure 11D).

### 3.5. Swainsonine

Notably, the symbiont did not colonize the outer margin of the *I. carnea* leaf surface. We suspected that swainsonine concentrations may differ between tissues that were colonized and not colonized on the adaxial surface of the leaf. Swainsonine concentrations did not differ between leaf tissues that were or were not colonized on the adaxial surface of the leaf.

## 4. Discussion

The study of plant–fungal interactions is a key component of understanding the evolutionary relationship between host and symbiont. In this study, micrographs illustrated no cellular damage due to the fungal colonization, and mycelia were detected growing in spaces (gaps) between different tissues or on the surface of different tissues both in the seed, 5-day-old seedlings, and mature plants. There was no evidence observed of the Chaetothyriales symbiont growing in between cells, as has been observed for *Alternaria* symbionts associated with *Astragalus* and *Oxytropis* species [19,20,21].

The micrographs reported herein support previous observations that the Chaetothyriales symbiont associated with *I. carnea* is seed transmitted [18]. The symbiont was detected by confocal, SEM, and FISH near the seed hilum, the structure at the tip of the funiculus where abscission occurs. The symbiont was also detected by confocal and SEM in hydrated seeds near the hypocotyl and the sclereids. We suspect as the seed germinates and is exposed to water and nutrients that this may serve as a trigger for symbiont to begin to grow and colonize the developing plant. The micrographs reported herein also show that the symbiont is associated with meristematic regions of five-day-old seedling and mature plants. The presence of the symbiont in these tissues provides a mechanism for the symbiont to colonize the newly developing plant as well as colonization of new growth after a plant is clipped back to woody stems.

Swainsonine has been detected in several *Ipomoea* species that have been reported to be toxic to different livestock species [7,8,9,25]. The Chaetothyriales symbiont was detected around the hilum in two of these species, *I. carnea* and *I. riedelii*. It is highly likely that other *Ipomoea* species that contain swainsonine are associated with their respective symbiont in a similar way. Notably, other species of the Convolvulaceae are associated with another distinct group of seed-transmitted fungal symbionts classified in the genus *Periglandula* (Hypocreales) [30] that produce the ergot alkaloids and indole diterpenes similar to those found in grasses infected with fungal endophytes from the same fungal family [31]. Microscopy has shown that this symbiont has a similar growth habit on the adaxial leaf surfaces only. It may be that the *Periglandula* symbiont will be detected in analogous parts of the seeds and meristematic regions of the plants in taxa containing the ergot alkaloids and/or indole diterpenes. These fungal symbionts, although different, may have a common evolutionary life history in how they are associated with the plant.

Trichomes are known to secrete large amounts of specialized metabolites that may provide resources for other organisms [32,33]. The close association of the host’s glandular trichomes and the Chaetothyriales symbiont in multiple tissues including the hypocotyl, the SAM, and the leaves may suggest that the symbiont benefits from the host due to some unknown factors secreted by the peltate glandular trichomes. This is consistent with the findings reported by Leistner et al. [33] investigating the association of *Periglandula* and the host *I. asarifolia*. These investigators concluded that the *Periglandula* symbiont may benefit from sesquiterpenes and fatty acids secreted by the host and that the ergot alkaloids produced by the symbiont are transported or passed into the host suggesting the trichomes are likely to have a dual and key function in a metabolic dialogue between symbiont and host plant.

In conclusion, the swainsonine-producing Chaetothyriales symbiont was detected in the seed tissues and meristematic tissues of seedlings and mature plants. The data herein provides further evidence that this symbiont is seed transmitted and how it may colonize developing tissues.

## Data Availability

Not applicable.

## Supplementary Materials

The following supporting information can be downloaded at: https://www.mdpi.com/article/10.3390/microorganisms10030545/s1, Figure S1: Fluorescent laser scanning confocal microscope images show fine threads of mycelia in the hypocotyl immediately above the radicle and under the cotyledons indicated by the arrow (A), and no evidence of mycelia in other regions of the hypocotyl (B), (C), and (D). Scale bar—100 μm, Figure S2: Fluorescent laser scanning confocal microscope of mycelia or lack thereof on surfaces of tissues from positive *Ipomoea carnea* plants. Bud sheath tangled around the peltate glandular trichomes indicated by arrows (A), seed coat (B), anther (C), and the vascular bundle (D). Scale bar—100 μm, Figure S3: Fluorescent laser scanning confocal microscope images illustrate the lack of mycelial invasion in all regions of the radicle of positive *Ipomoea carnea* plants. Scale bar—100 μm.
