# Supplementary material for: Localization of the Swainsonine-Producing Chaetothyriales Symbiont in the Seed and Shoot Apical Meristem in Its Host Ipomoea carnea
Source: Microorganisms. 2022 Mar 2;10(3):545. doi: 10.3390/microorganisms10030545 (PMC8951018; doi:10.3390/microorganisms10030545)

**Supplemental Figures.**

Figure S1: Fluorescent laser scanning confocal microscope images show fine threads of mycelia in the hypocotyl immediately above the radicle and under the cotyledons indicated by the arrow (A), and no evidence of mycelia in other regions of the hypocotyl (B), (C), and (D). Scale bar- 100 µm.

Figure S2: Fluorescent laser scanning confocal microscope of mycelia or lack thereof on surfaces of tissues from positive *Ipomoea carnea* plants. Bud sheath tangled around the peltate glandular trichomes indicated by arrows (A), seed coat (B), anther (C), and the vascular bundle (D). Scale bar- 100 µm.

Figure S3: Fluorescent laser scanning confocal microscope images illustrate the lack of mycelial invasion in all regions of the radicle of positive *Ipomoea carnea* plants. Scale bar- 100 µm.

Supplemental Figure S1.

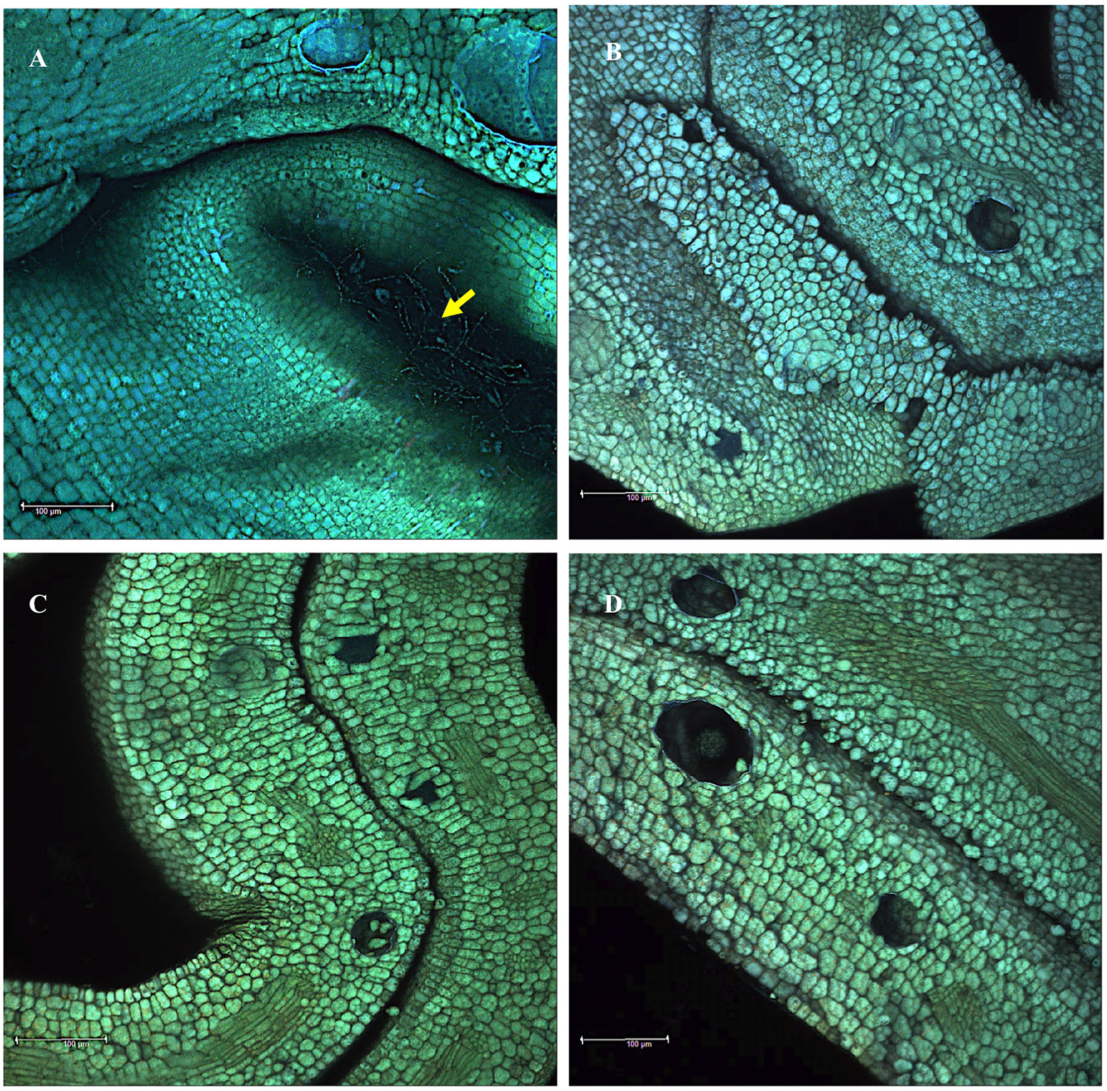

Supplemental Figure S2.

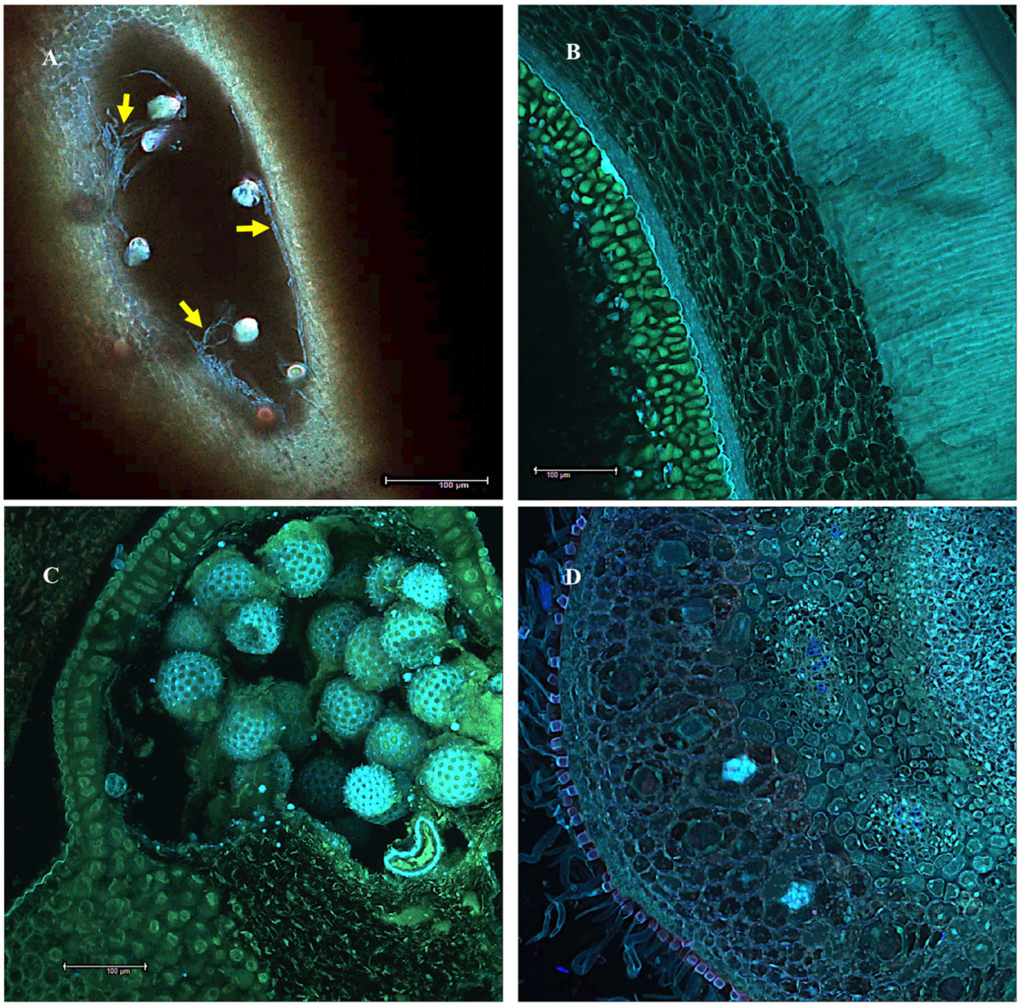

Supplemental Figure S3.

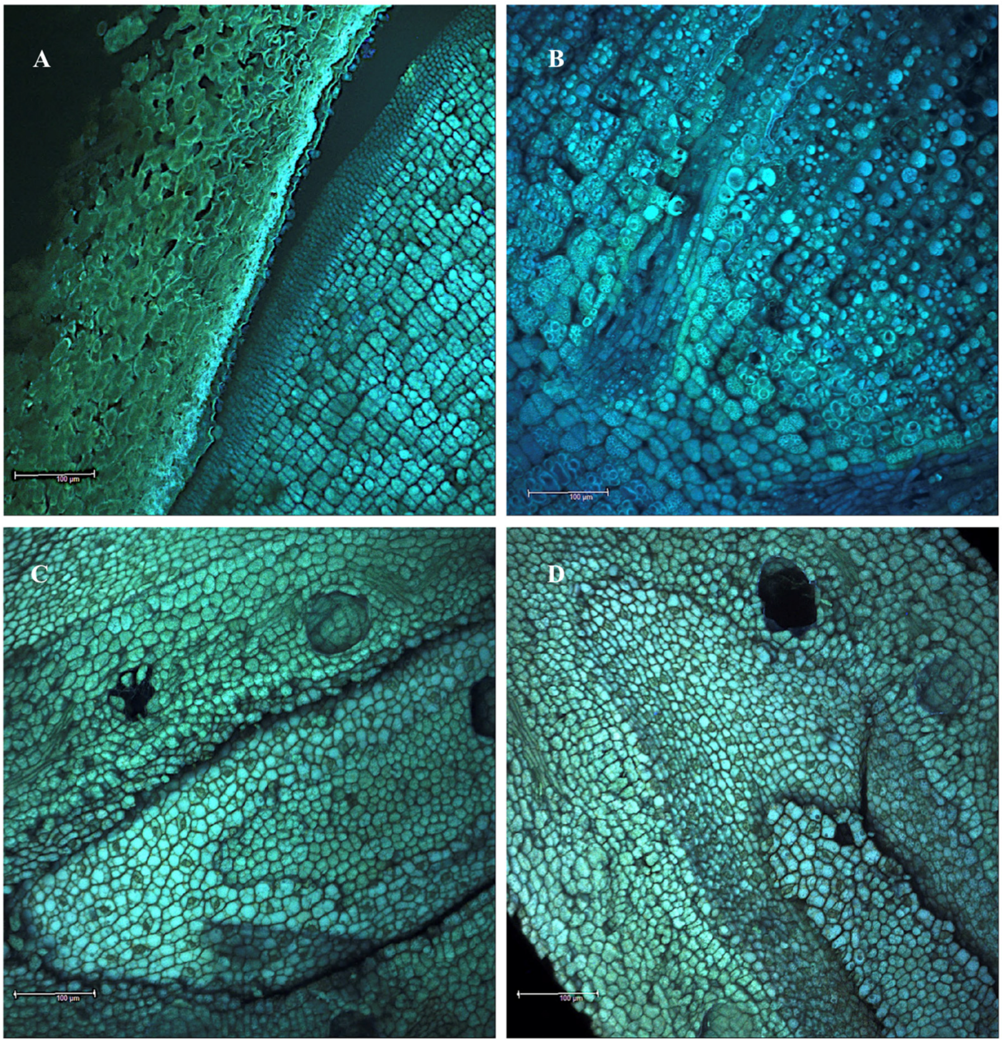

Supplement: Supplementary file 1 [file microorganisms-10-00545-s001.zip › microorganisms-1619739-supplementary-.pdf]
